# Personalizing non-small cell lung cancer treatment through patient-derived xenograft models: preclinical and clinical factors for consideration

**DOI:** 10.1007/s12094-024-03450-3

**Published:** 2024-03-29

**Authors:** Vered Fuchs, Ariel Sobarzo, Maha Msamra, Yarden Kezerle, Liat Linde, Gur Sevillya, Alaa Anoze, Yael Refaely, Ahron Yehonatan Cohen, Israel Melamed, Amit Azriel, Rami Shoukrun, Yael Raviv, Angel Porgador, Nir Peled, Laila Catalina Roisman

**Affiliations:** 1 The Shraga Segal Department of Microbiology, Immunology and Genetics, Faculty of Health Sciences, Ben-Gurion University of the Negev, Beer Sheva, Israel; 2 The Oncology Institute, Helmsley Cancer Center, Precision Oncology and Innovation, Shaare Zedek Medical Center, 12, Shmuel Beit St, 9103102 Jerusalem, Israel; 3 Institute of Pathology, Soroka University Medical Center, Beer-Sheva, Israel; 4 Biomedical Core Facility, Russell Berrie Nanotechnology Institute, Technion-Israel Institute of Technology, Haifa, Israel; 5 Department of Cardiothoracic Surgery, Soroka University Medical Center, Beer-Sheva, Israel; 6 The Oncology Institute, Soroka University Medical Center, Beer-Sheva, Israel; 7 Pulmonary Institute, Soroka University Medical Center, Beer-Sheva, Israel; 8 Department of Neurosurgery, Soroka University Medical Center, Beer Sheva, Israel; 9 Department of Ears, Nose & Throat, Head & Neck Surgery, Soroka University Medical Center, Beer Sheva, Israel

**Keywords:** Non-small cell lung cancer, Precision medicine, Preclinical models, Patient-derived xenografts, NSG-SGM3

## Abstract

**Purpose:**

In the pursuit of creating personalized and more effective treatment strategies for lung cancer patients, Patient-Derived Xenografts (PDXs) have been introduced as preclinical platforms that can recapitulate the specific patient’s tumor in an in vivo model. We investigated how well PDX models can preserve the tumor’s clinical and molecular characteristics across different generations.

**Methods:**

A Non-Small Cell Lung Cancer (NSCLC) PDX model was established in NSG-SGM3 mice and clinical and preclinical factors were assessed throughout subsequent passages. Our cohort consisted of 40 NSCLC patients, which were used to create 20 patient-specific PDX models in NSG-SGM3 mice. Histopathological staining and Whole Exome Sequencing (WES) analysis were preformed to understand tumor heterogeneity throughout serial passages.

**Results:**

The main factors that contributed to the growth of the engrafted PDX in mice were a higher grade or stage of disease, in contrast to the long duration of chemotherapy treatment, which was negatively correlated with PDX propagation. Successful PDX growth was also linked to poorer prognosis and overall survival, while growth pattern variability was affected by the tumor aggressiveness, primarily affecting the first passage. Pathology analysis showed preservation of the histological type and grade; however, WES analysis revealed genomic instability in advanced passages, leading to the inconsistencies in clinically relevant alterations between the PDXs and biopsies.

**Conclusions:**

Our study highlights the impact of multiple clinical and preclinical factors on the engraftment success, growth kinetics, and tumor stability of patient-specific NSCLC PDXs, and underscores the importance of considering these factors when guiding and evaluating prolonged personalized treatment studies for NSCLC patients in these models, as well as signaling the imperative for additional investigations to determine the full clinical potential of this technique.

**Supplementary Information:**

The online version contains supplementary material available at 10.1007/s12094-024-03450-3.

## Introduction

Lung cancer (LC) is the most common cause of cancer-related mortality worldwide [1]. Up to 85% of lung cancer cases are non-small-cell lung cancer (NSCLC), which often presents an advanced course of disease related to poor survival rates [2]. In the past two decades, targeted molecular therapies and immunotherapies, specifically immune checkpoint inhibitors (ICIs), have become the backbone of care for NSCLC patients and have dramatically improved outcomes [3–5]. The benefit of ICI treatment primarily stems from the ability to identify biomarkers and tailor specific treatments accordingly. [3]. Despite the significant benefits, in advanced NSCLC, only ~ 20–30% of patients respond to treatments [6, 7]. Even among responders, these treatments may have serious drawbacks, including significant side effects and, in rare cases, aggravation of the course of the disease, causing what is known as "hyperprogression" [8]. The questions of whether a patient will respond to treatment and how the treatment will affect an individual remain complex and challenging to resolve and involve intricate factors, including the tumor, host, and environmental factors, as well as their synergistic action [4]. It has long been known that cancerous tumors differ in their genetic profiles; however, advances in cancer research, including the use of innovative technologies such as next-generation sequencing (NGS), have elucidated the role of intratumor heterogeneity (ITH) [9]. Genetic heterogeneity and tumor plasticity allow cancer cells to survive treatment and lead to drug resistance and tumor metastasis, two main causes of mortality [10].

Thus, the need for personalized predictive preclinical models that can accurately represent the tumor, allowing to prophesy the response of a specific patient to be improved, is increasing. Patient-derived xenograft (PDX) mouse models have gained popularity. They have been used as an in vivo models in numerous cancer types, including lung cancer [11, 12], breast cancer [13], colorectal cancer [14], pancreatic cancer [15], prostate cancer [16], ovarian cancer [17], and CNS tumors [18]. PDX models are generated by the implantation of surgically resected tumor specimens, either primary tumors or metastases, typically into immunodeficient mice [19, 20]. The transplanted tumor is usually serially transferred throughout numerous generations of mice, referred to as passages, allowing to amplify the tissue biopsy and retain it in an in vivo model continuously. PDX models have been known to recapitulate the tumor’s histological and molecular characteristics, offering a superior alternative to the conventional in vitro cell culture models for cancer research and drug screening [21]. However, the potential of the PDX model to serve as a "true" preclinical tool has been questioned [21, 22]. Recent studies have shown high variation in PDX engraftment success, growth kinetics, and tumor stability [23]. Several factors have been shown to affect PDX engraftment, including biopsy quality, time to transplant, patient treatment history, and disease stage [12, 24]. In addition, it has also been suggested that host factors like the murine stroma interfere with the human tumor-associated stroma, thus hampering the results of genetic analysis and tumor microenvironment (TME) [22]. Indeed, research groups have shown that PDXs undergo mouse-specific genetic evolution that differs from the tumor evolution nature in humans [25, 26].

One of the main challenges of the PDX model is its ability to reflect the tumor's properties faithfully throughout serial passages in mice [21, 25, 27] to enable extensive study duration. Such a model is critical, particularly in biopsies collected from patients with advanced progressive disease, likely showing treatment resistance.

In this study, we developed and established an NSCLC PDX model in NSG-SGM3 mice. We assessed multiple clinical and preclinical factors influencing PDX engraftment success, growth kinetics, and tumor stability. We further performed histopathological and molecular analyses to understand the model's capability to reflect tumor heterogeneity throughout serial passages. We hypothesized that PDXs may not accurately represent the tumor's clinical and molecular features across sequential passages and that various clinical and preclinical factors affect the propagation of patient-derived xenografts in NSCLC models.

## Materials and methods

### Patients and tissue specimens

Fresh tumor specimens (40 numbers) were obtained from NSCLC patients who underwent surgical removal at Soroka Medical Center, Beer-Sheva, Israel, between 2018 and 2021. After surgery, tumor specimens were sent for pathological assessment and subsequent confirmation and aliquoted for DNA extraction, cryopreservation, and implantation in NSG-SGM3 mice. The specimen process was performed within 2 h following surgical extraction. Written informed consent was obtained from all patients. The ethics committee of Soroka Medical Center (No. 0026-19-SOR) approved this study.

### Animals

Non-obese diabetic/severe combined immune deficiency (NOD/SCID) (4–5-week-old) triple transgenic NSG-SGM3 mice were obtained from the Jackson Laboratory (Sacramento, CA, USA). Animals were housed in individually ventilated cages at an appropriate temperature (21–25 °C) with a 12-h light/dark cycle and free access to food and water. All experiments and protocols in mice were approved by the Institutional Animal Care and Use Committee of BGU (authorization number IL-59-11-2018-(A), and the Israel Ministry of Health (MOH). All experiments were performed in accordance with relevant guidelines and regulations. This study is reported in accordance with the ARRIVE guidelines.

### PDX model establishment

PDX models were generated as previously described with minor modifications [19, 20]. Briefly, fresh tumor specimens (up to 2 h after surgical restriction) were divided into fragments of approximately 2–3 mm^3^ and implanted subcutaneously into the flanks of 6–7-week-old NSG-SGM3 mice. Each patient specimen was implanted into 3 NSG-SGM3 mice, which were then monitored for tumor growth for up to 180 days. When the tumor size was > 1000 mm^3^, the PDX mice were anesthetized with carbon dioxide, and the tumors were surgically removed. Extracted tumor tissue was used for passaging, DNA extraction, and histopathological examination.

### Histological staining

Histological staining of tumor biopsies and PDXs was done as follows: 4 μm sections of the sample were fixed in 4% formaldehyde and embedded in paraffin using an automated staining device (Benchmark XT; Ventana Medical Systems, Tucson, AZ, USA). For hematoxylin and eosin (H&E) staining, a pathologist reviewed the pathological type and architecture between the original and xenograft tumors to confirm the diagnosis.

Sample sections were also incubated with the following antibodies: anti-Ki-67 (1:400 dilution; Abcam, Cambridge, UK); anti-TTF1 (Transcription Termination Factor 1) (1:200 dilution; Cell Signaling Technology, Danvers, MA, USA); anti-P40 (1:100 dilution; Cell Signaling Technology); and anti-PD-L1 (programmed death-ligand 1) (1:100 dilution; Abcam) to compare the immunophenotypic characteristics. Images were acquired using a Nikon Eclipse Ci microscope with a Nikon DS-Fi3 camera and NIS-elements software, version 5.21.00. All procedures were done by a medical pathologist in Soroka Medical Center.

### Whole exome sequencing (WES)

DNA was extracted from 8 PDX tissue samples using the QIAcube Connect (Qiagen) with the DNeasy blood and tissue kit according to manufacture instructions (Cat No. 69504). Briefly, tissues were thawed to room temperature, and a piece of ~ 25 mg was cut and placed in a 1.5 ml tube. 180ul ATL buffer and 4 0ul of proteinase K were added to the tissue, and the samples were incubated on a thermomixer at 56 °C, 1000RPM overnight. The samples were then centrifuged at 3 °C at 10,000 × g, and the supernatant was used for the extraction. DNA concentration and quality were evaluated using NanoDrop One (Thermo Scientific). Next, 8 exome libraries were constructed simultaneously according to the manufacture protocol (Illumina DNA Prep with Enrichment, Illumina, Cat No. 20025523) using 700 ng DNA as a starting material. After the amplification of fragmented DNA, the quality of the libraries was determined using the TapeStation 4200 with the High Sensitivity D1000 kit (Agilent Cat No. 5067–5584), and the concentration of the libraries was measured using the Qubit dsDNA HS Assay Kit (Invitrogen, Cat No. Q32851). The libraries were then pooled together for the enrichment stage (200 ng per sample). The enrichment was performed using the xGen™ Exome Research Panel v2 (IDT 4 rxn kit). After the enrichment, the concentration of the library was measured using the Qubit dsDNA HS Assay Kit (Invitrogen, Cat No. Q32851), and the size was determined using the TapeStation 4200 with the High Sensitivity D1000 kit (Agilent Cat No. 5067–5584). Data generation was performed on the Illumina NextSeq2000, P2 200 cycles (Read1-101; Read2-101; Index1-10; Index2-10) (Illumina, Cat No. 20046812). Quality control was assessed using Fastqc (v0.11.8). Reads were trimmed for adapters, low quality 3`, and a minimum length of 20 using CUTADAPT (v1.10). The 100 bp paired-end reads were aligned to the human genome. The reference genome and known SNPs and INDELs databases for genome version hg38 (required by GATK) were downloaded from the GATK bundle.

### Statistical analysis

Statistical analyses were performed using GraphPad Prism 8.4.2. To determine clinical parameters that contributed to the establishment of the PDXs, logistic regression analysis was conducted to evaluate the correlation between the success rates and the clinical, demographic, and pathological parameters. A value of P < 0.05 was considered statistically significant. Statistical methods are indicated in the text and figure legends where applicable.

## Results

### Establishment of an NSCLC PDX mouse model platform in NSG-SGM3 mice

**Fig. 1:**
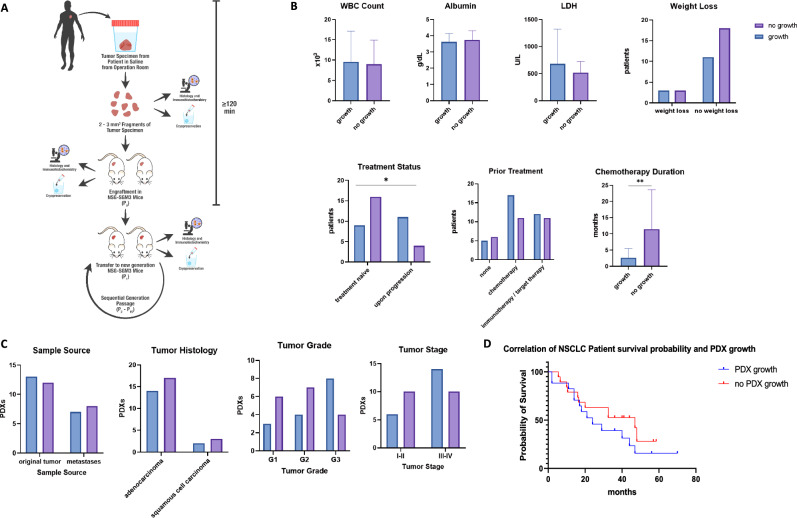
Establishment of the NSCLC PDX model platform **A** Schematic presentation of PDX engraftment protocol in NSG-SGM3. Tumor tissues were collected from NSCLC donor patients undergoing surgical tumor removal after obtaining informed consent. Following resection, the tumor biopsy was kept in a saline solution and transferred to the pathology lab for examination. After pathology confirmation, within 120 min, the biopsy was dissected into smaller fragments of 2–3 mm^3^ and implanted subcutaneously into the tissue back or flank area of NSG-SGM3 mice, thus creating the first generation of Patient-Derived Xenograft (PDX), referred to as P0 (passage 0). The remaining tumor tissue was cryopreserved for genetic analysis and assessed for pathology and immunohistochemistry. Once a formed PDX reached the size of 1000mm^3^, mice were euthanized, and the tumor was dissected into smaller implant fragments of 2–3mm^3^. Subsets of these were as follows: (1) transferred into a new generation of NSG-SGM3 mice (P1–P10), (2) cryopreserved for genetic analysis and sample bank collection, and (3) assessed for pathology and immunohistochemistry. The process repeated sequentially throughout multiple passages of PDX mice. **B** Clinical and demographic parameters of NSCLC donor patients. The patient's demographic and clinical data were collected at the tumor excision and PDX engraftment point. PDX engraftment was analyzed against the following parameters: WBC count, albumin levels, LDH levels, weight loss > 10% from diagnosis of disease until engraftment of PDX, treatment history, types of treatments, and chemotherapy treatment duration. Data are shown as mean results. Error bars represent standard deviation (SD). **C** Pathological features of NSCLC PDXs. Several pathological features were analyzed during surgical tumor excision and correlated to PDX growth success and growth rate. These included sample source, tumors' histological type, tumors' histological differentiation grade, and tumors' histological staging according to TNM classification for NSCLC. **D** Correlation of NSCLC patient survival probability and PDX growth. PDX growth was correlated with the survival time of corresponding patients. Kaplan–Meier survival curves are shown. The tick marks represent censoring

To study tumor stability and develop a preclinical platform for the treatment evaluation of NSCLC patients, we first established a PDX model platform in NSG-SGM3 mice. NSG-SGM3 mice were selected for our models due to their ability to engraft both myeloid and lymphoid-derived cells stably, thus providing a superior alternative to their backbone, commonly used NSG strain for humanization models. Fresh PDX samples from NSCLC donors were collected during surgical resections and, after pathological confirmation, engrafted in NSG-SGM3 mice. Transplant procedures were performed within two hours of biopsy collection. A total of 40 patient PDXs were engrafted, 20 of which successfully grew and formed propagating tumors in mice after 80 weeks, for a tumor engraftment rate of 50%. Successful PDX transplants were serially passaged in mice to create a "hot" sample bank and aliquoted and cryopreserved for histopathological and molecular analysis. A schematic presentation of the process is presented in Fig. 1A.

### Patient demographics and clinical features

**Fig. 2:**
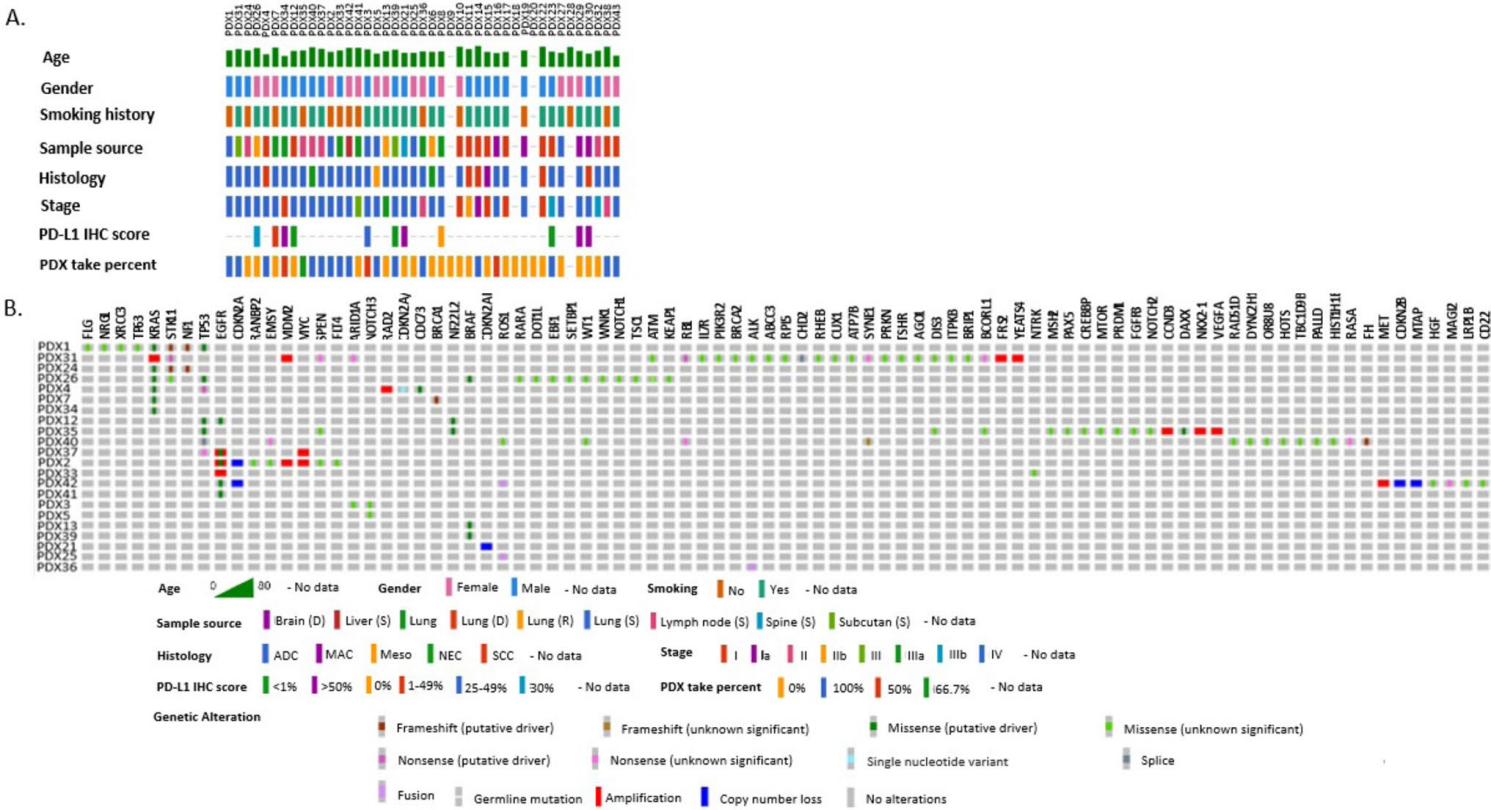
Oncoplot presenting the clinical background and molecular alterations of each NSCLC PDX patient. **A** The plot displays the age, gender, smoking history, PDX sample source, tumor histology, tumor stage, PDL-1 immunohistochemistry score, and the PDX take percent within engrafted mice. The PDX take percent represents the percentage of PDXs successfully engrafted in the mice. PDL-1 immunohistochemistry score is indicated in percentage ranges. **B** The plot displays alterations found by clinical liquid or tissue sequencing panels for each patient. Alterations are color coded based on their type (e.g., amplifications, deletions, missense mutations). The alterations identified by clinical sequencing panels are shown on the upper side of the plot

To study the correlation between PDX engraftment success rates and patient clinical characteristics, we obtained demographic and clinical data from our cohort of NSCLC donor patients (presented in Table 1). Our data showed that the ratio of males to females was 23:17, and the average age of patients was 62.6 years, ranging from 42 to 80 years. Twenty-seven (67.5%) patients had smoked regularly in the past. The tumor histologic types included adenocarcinoma (*n* = 27), squamous cell carcinoma (*n* = 6), neuroendocrine carcinoma (*n* = 1), and other histological types (*n* = 6). Samples were resected from lesions in the lung (*n* = 25), lymph nodes (*n* = 5), brain metastasis (*n* = 6), subcutaneous lesions (*n* = 2), spine (*n* = 1), and liver (*n* = 1). Figure 2 displays the clinical features of each participant, including the molecular alterations detected by clinical sequencing panels (if applicable) and the corresponding engrafted PDX take rate. Detailed information about each patient’s clinical background and treatment history are presented in Table S1.

**Table 1:** Patient clinical characteristics and NSCLC tumor features

Measure	Total (%)	PDX growth (%)	No PDX growth (%)	*p* value
Total	40 (100%)	20 (50%)	20 (50%)	
Gender				0.7491
Male	23 (57.5%)	11 (27.5%)	12 (30%)	
Female	17 (42.5%)	9 (22.5%)	8(20%)	
Age				0.9615
Average	62.625	62.55	62.7	
Range	42–80	42–80	51–77	
Smoking history				0.3112
Yes	27 (67.5%)	12 (30%)	15 (37.5%)	
No	13 (32.5%)	8 (20%)	5 (12.5%)	
Sample source				0.8294
Lung	26 (65%)	13 (32.5%)	13 (32.5%)	
Lymph nodes	5 (12.5%)	3 (7.5%)	2 (5%)	
Brain	5 (12.5%)	1 (2.5%)	4 (10%)	
Subcutaneous	2 (5%)	2 (5%)	0 (0%)	
Spine	1 (2.5%)	0 (0%)	1 (2.5%)	
Liver	1 (2.5%)	1 (2.5%)	0 (0%)	
Histologic type				
Adenocarcinoma	30 (75%)	14 (35%)	16 (40%)	
Squamous cell carcinoma	5 (20%)	2 (5%)	3 (7.5%)	
Neuroendocrine carcinoma	1 (2.5%)	0 (0%)	1 (2.5%)	
Other	4 (10%)	3 (7.5%)	1 (2.5%)	
Differentiation grade				0.1136
G1	9 (22.5%)	3 (7.5%)	6 (15%)	
G2	11 (27.5%)	4 (10%)	7 (17.5%)	
G3	12 (30%)	4 (10%)	8 (20%)	
Unknown	8 (20%)	4 (10%)	4 (10%)	
Staging				0.332
I–II	16 (40%)	6 (15%)	10 (25%)	
III–IV	24 (60%)	14 (35%)	10 (25%)	
Prior treatment				0.7062
Early-stage resectable disease	25 (62.5%)	9 (22.5%)	16 (40%)	0.0484
None	13 (32.5%)	6 (15%)	7 (17.5%)	
Neoadjuvant chemotherapy	9 (22.5%)	7 (17.5%)	2 (5%)	
TKI	3 (7.5%)	0 (0%)	3 (7.5%)	
Advanced disease/recurrence	15 (37.5%)	11 (27.5%)	4 (10%)	0.0484
Chemotherapy	14 (35%)	11 (27.5%)	3 (7.5%)	
Immunotherapy	12 (30%)	9 (22.5%)	3 (7.5%)	
TKI	4 (10%)	0 (0%)	4 (10%)	
ALK inhibitor	1 (2.5%)	0 (0%)	1 (2.5%)	
Radiation therapy	6 (15%)	5 (12.5%)	1 (2.5%)	
Chemo treatment duration				
Average (months)	6.85	2.83	12.08	0.0368

### Factors affecting the success rate of initial engraftment

Specific patient clinical features were compared to the engraftment ability of their corresponding PDXs to understand which parameters may affect successful PDX growth. We evaluated clinical metrics, including specific laboratory results on the day of tumor resection, treatment history, and tumor histopathological characteristics. We found no correlations between engraftment and patient levels of white blood cells (WBC count), albumin, and LDH (Fig. 1B). Nor was there a difference in engraftment success between patients who exhibited noticeable weight loss and those who did not. Patients with early-stage resectable tumors were less likely to form PDXs, compared to tumors from patients on advanced treatment lines following progression at the time of resection (Fig. 1B). In addition, a negative correlation was seen between prior chemotherapy treatment duration and engraftment success (Fig. 1B).

We further examined the relationship between tumor pathological characteristics and the rate of tumor engraftment in the PDX model. We did not find a significant difference between samples from the primary tumor and those from metastatic sites (Fig. 1C). Additionally, the histological subtypes did not affect tumor engraftment success rates (Fig. 1C). However, an advanced stage or grade of the disease was linked to higher chances of the successful growth of the PDX (Fig. 1C). Finally, we analyzed the overall survival of all patients and found that the probability of survival was lower among patients with successful PDX growth (Fig. 1D). The specific PDX take rates per patient are detailed in Table S1.

### Factors affecting PDX growth kinetics

**Fig. 3:**
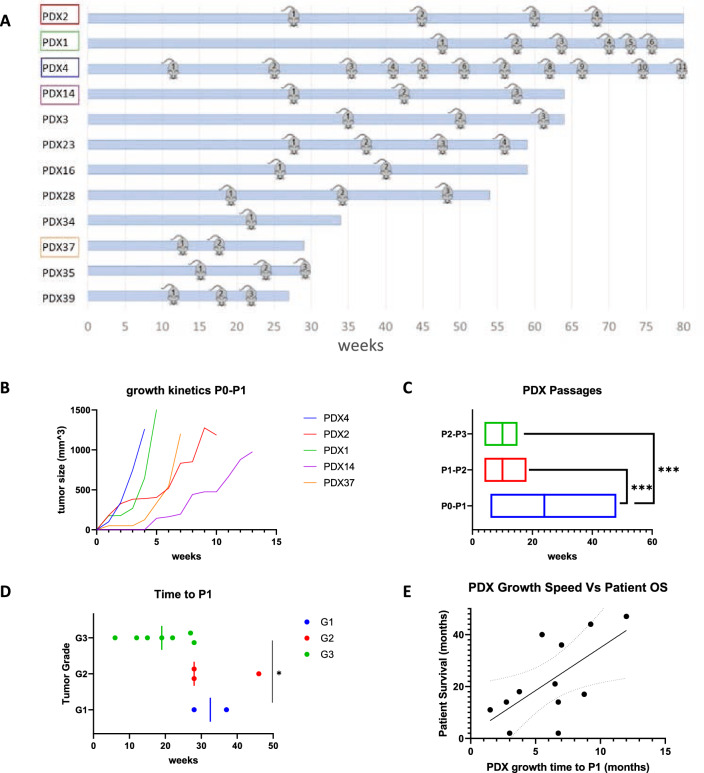
Growth patterns of NSCLC PDX grafts in NSG-SGM3 mice. Following the engraftment of tumors in mice, PDX size was measured regularly, and once the xenograft reached the size of 1000 mm^3^, mice were euthanized. The PDX was serially transferred to the next generation of mice. **A** Passage patterns of 13 selected NSCLC PDXs engrafted in NSG-SGM3 mice over 80 weeks. Each bar represents the time from the initial engraftment of the PDX. Mouse icons represent the passage of the PDX to a new generation of NSG-SGM3 mice. The passage number is indicated inside the mouse icons. **B** Growth kinetics of 5 PDXs in NSG-SGM3 mice throughout 15 weeks from engraftment (P0) to first passage (P1). **C** Time intervals between engraftment of PDXs to first passage (P0–P1), first passage to second passage (P1–P2), and second passage to third passage (P2–P3). Lines represent the median results. **D** Correlation of first PDX passage duration time (P0–P1) and tumor histological differentiation grade. Lines represent median results. **E** Relationship between patient overall survival and corresponding PDX duration time to the first passage

Successfully engrafted NSCLC PDXs were sequentially passaged up to 10 times (P10) in NSG-SGM3 mice, and tumor size was measured twice weekly to study PDX growth kinetics. Growth rates varied between the engrafted tumors, creating different time intervals for passing each PDX, as presented in Fig. 3. Diverseness in growth rates was associated with the implanted tumor's clinical behavior, indicating a positive correlation between the tumor aggressiveness and its ability to propagate successfully in mice and grow more rapidly.

Diverseness was most prominent in the time interval from implantation of the original tumor until the first passage, referred to as P1. This time interval includes the latency period, the time from tumor transplantation until the primary initiation of PDX propagation. Representative growth curves of 5 tumors from initial PDX growth in mice until the first passage are shown in Fig. 3B. Overall, the median time to the first passage was 24.0 weeks. In contrast, the second passage was required after a median period of 10.0 weeks, and a third passage was needed. A statistically significant difference was observed between the first passage and the following two passages (*P* value = 0.005, shown in Fig. 3C). Additionally, a significantly shorter interval to the first passage was observed in tumors of higher differentiation grades (Fig. 3D). Finally, a negative correlation was found between PDX growth speed until P1 and overall patient survival, as shown in Fig. 3E.

### Pathological and genomic consistency during PDX passages

**Fig. 4:**
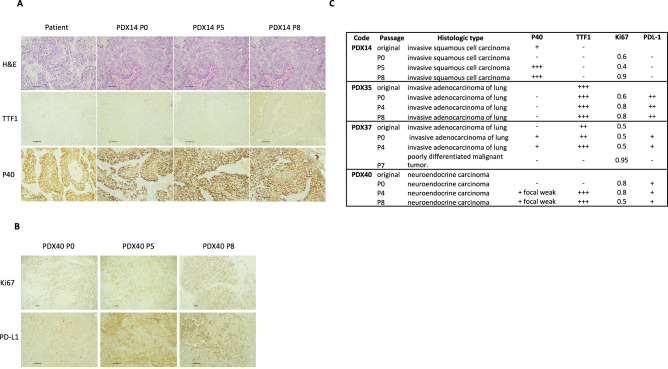
Immuno-histological staining characterization of NSCLC PDXs. PDXs were stained for hematoxylin and eosin (H&E) and analyzed for tumor structure and pathological clinical markers, including TTF1, P40, Ki67, and PDL1. **A** Representative H&E, TTF1, and P40 staining results are shown for PDX14 in the patient tumor, early (P0), intermediate (P5), and late (P8) PDX passages. **B** Representative Ki67 and PD-L1 staining results are shown for PDX40 in early (P0), intermediate (P5), and late (P8) PDX passages. **C** Immuno-histological staining results of selected NSCLC PDXs. Histopathology images were acquired at a magnification of X100 for the hematoxylin & eosin, TTF-1, p40, and PD-L1 slides and at a magnification of X40 for Ki67 slides

To determine whether NSCLC PDXs retain their original tumors' histopathological characteristics and if these are preserved throughout generations of mice, the original tumor and corresponding PDXs from serial passages were analyzed for histopathological features. Each tissue was stained for hematoxylin, eosin (H&E), and related clinical markers, including TTF1, P40, Ki67, and PDL1. The periodic histopathological results of the 4 selected PDXs are shown in Fig. 4.

We found that the histologic type and differentiation grade of NSCLC PDXs throughout mouse generations were preserved and remained stable compared with those of the initially engrafted tumor. Quantification of molecular biomarkers using standard scoring algorithms revealed few changes between tested biomarkers and PDX passages (Fig. 4C). The levels of the markers TTF1, Ki67, and PDL-1 were generally steady in the early passages of all PDXs; however, the levels were slightly altered in advanced passages (P7-8). Marker P40 showed the highest variation between the different passages of PDXs compared to the original tumor for PDX14 and PDX37.

**Fig. 5:**
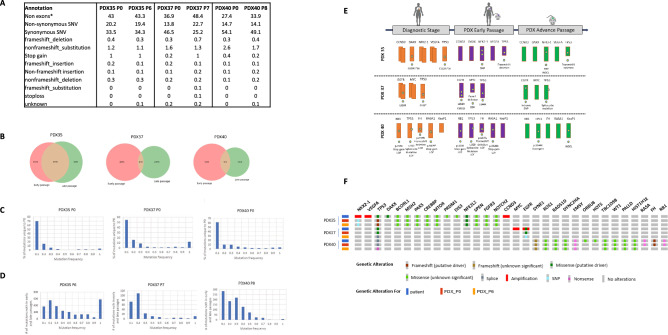
Whole exome sequencing (WES) analysis. Selected NSCLC PDXs from paired early and late corresponding passages were analyzed by WES. **A** Annotation distribution (%) of passed filter variants according to WES analysis for selected NSCLC PDXs from early and corresponding late passages. **B** Venn diagram representing shared variants across early and late passages of paired PDXs. **C** The distribution of frequencies of passed filter variants occurred only in early PDX passages. **D** The distribution of passed filter variants occurred both in the early and in the corresponding late passages of NSCLC PDXs. **E** Comparison of clinically relevant variants found in diagnostic NSG performed for NSCLC patients before tumor resection and WES analysis results of the corresponding early and late PDX passages. Clinically relevant genes are schematically represented as rectangles; gene names are presented above each rectangle; gene alterations are described as stars; and the specific mutation is noted below each rectangle. A double rectangle represents gene amplification; a shortened rectangle represents gene deletion or copy number loss. **F** Oncoplot showing the molecular alterations of three NSCLC PDXs throughout sequential passages. Each row represents a single PDX sample, with the patient ID on the left. The columns indicate different molecular alterations detected by clinical sequencing panels at the original tumor and by whole exome sequencing in the first passage (P0) and advanced passages (P6–P8). Alterations are color coded based on their type (e.g., amplifications, deletions, missense mutations)

In the next step, we aimed to determine the genomic fidelity of NSCLC PDXs throughout sequential passages. Whole exome sequencing (WES) was performed on selected PDXs at their first passage (P0) and an advanced passage (P6–P8). The representative results of three PDXs, PDX35, PDX37, and PDX40, are presented in Fig. 5. The genomic analysis showed 864, 485, and 907 non-synonymous SNVs detected in the first passaged PDX35, PDX37, and PDX40 xenografts, respectively. These included a maximum of 20 frameshift deletions in PDX40 P0, 10 frameshift insertions in PDX35 P0, 44 stop gain mutations seen in PDX35 P0, and a maximum of 1 stoploss mutation in PDX40 P0. In the advanced passaged PDXs, 910, 594, and 543 non-synonymous SNVs were detected in PDX 35 P6, PDX37 P7, and PDX40 P8, respectively, with a maximum of 19 frameshift deletions in PDX37 and a maximum of 7 frameshift insertions, 45 stop gain mutations, and 2 stop loss mutations all seen in PDX 35. Annotation distribution is presented in Fig. 5A.

Frequency analysis of the variants identified in the PDXs found that most alterations were rare and were detected in a frequency of less than 0.1. These results were consistent in all PDXs sequenced in their early and late passages. Paired analysis of early and late passage PDXs showed that only PDX35 retained a prominent number of alterations across the early and late passages consisting of 2735 matching mutations. In contrast, PDX37 and PDX40 had minimal similarity between their early and advanced passage samples, with 245 and 942 coequal variants, respectively (Fig. 5B). Although PDXs showed, in general, low-frequency variants, analysis of alterations that occurred in both early and late passages found higher frequency variations (Fig. 5C, D).

We checked whether specific target mutations were retained throughout PDX generations to gain a more clinically relevant perspective. As part of personalized treatment, molecular analyses using liquid or tissue biopsies were performed for all patients in various stages of the disease to detect clinically relevant mutations and follow tumor genomic dynamics. The molecular results of these panels were compared with the genomic results of PDXs' early and late passages.

The comparison analysis presented in Fig. 5E, F shows clinically relevant mutations in PDX35 in the diagnostic stage before tumor resection, including CCND3 amplification, DAXX C629fs*16, NKX2-1 amplification, TP53 F212fs*34, and VEGFA amplification. Only NKX2-1 and TP53 mutations were seen in the early and late passages of the engrafted PDX. As for PDX37, liquid biopsy sequencing found EGFR L858R, EGFR amplification, TP53, and MYC amplification. EGFR L858R mutation was found in the early corresponding PDX in addition to a new EGFR mutation found in exon 25. In the late PDX passage, only an intronic EGFR mutation was detected. TP53 mutations were present in the early and late PDX passages with different nucleotide variants. PDX40 analysis at the diagnostic stage comprised RB1 stop gain mutation, TP53 splice site variant, RASA1 stop gain mutation, and FH frameshift mutation, which all remained in the early but not in the late PDX generation, and KeaP1 mutations, which were not found in the early PDX generation, but a different mutation variant was found in the late PDX generation. TP53 stop gain mutation was seen in the late PDX generation. In addition, several mutations of unknown significance were detected in the clinical setting in PDX35 and PDX40, and they mainly were retained in the early (P0) and in the advanced (P6-8) PDX samples as shown in Fig. 5F.

## Discussion

NSCLC PDX mice models have been developed and are used as a preclinical platform to guide personalized treatment decisions for NSCLC patients. However, their capacity to mirror tumor characteristics, especially in the advanced stage and throughout the long-term models of serial passages, is still inconclusive. To address this, we developed an NSCLC PDX NSG-SGM3 mouse model, studied tumor stability, and evaluated growth kinetics. We analyzed preclinical and clinical factors against engraftment success throughout multiple generations of PDXs. We have chosen to use NSG-SGM3 immunodeficient mice as hosts for our NSCLC PDX model. To date, only minimal data are available using NSG-SGM3 mice as a model for solid tumors and none to our knowledge for NSCLC. Given the strain's ability to develop a broader spectrum of human immune cells, it holds the potential to be used as a central platform for understanding the human immune system and tumor microenvironment interactions in a "human setting." These traits may be leveraged to create a humanized NSCLC mouse model that mimics the patient's tumor and immune cell population, providing a superior preclinical platform for immuno-oncology efficacy studies.

Following our model development, tumor aggressiveness, reflected by an advanced stage and grade of NSCLC, was associated with higher PDX engraftment success rates and faster PDX growth. A correlation was found between PDX growth speed in the first generation and overall patient survival, suggesting the model may be a valuable predictor of patient prognosis. Surprisingly, no relation was found between several clinical factors related to the prognosis and survival of patients, including severe weight loss, LDH levels, albumin levels, and WBC counts. This lack of correlation may be attributed to the time of sample collection. These measurements were documented proximate to tumor resection and may have been altered by prior treatment exposure or various temporal effects. Concerning patient treatment history, our study showed that naïve patients and patients undergoing extended chemotherapy treatments were less likely to form successful PDXs. These findings emphasize the importance of proper patient selection, considering treatment history for developing and using in vivo PDX models, and are in accordance with other works [11, 12].

PDX growth kinetic results showed great diversity between the various PDXs, reflecting tumor heterogeneity. We found that PDXs, such as PDX4 and PDX1, were fast-growing tumors requiring passage every 3–10 weeks following the latency period (Fig. 3A, B). In contrast, slow growth kinetics were observed with PDX3, PDX14, and PDX28, requiring passage every 7–15 weeks. Latency periods were usually concordant with the PDX growth patterns following initial growth, besides PDX1, which interestingly had an extended latency period of 48 weeks. However, once it started to thrive, it showed a rapid growth curve requiring frequent passaging.

Additional analysis between growth kinetics and disease progression in the clinic showed a strong correlation; for example, PDX4, a fast-growing tumor, was clinically highly aggressive, and led to the patient's death after only 10.6 months from diagnosis. PDX14, on the other hand, was a slow-growing PDX. The patient had a relatively indolent disease with overall survival of 18.5 months.

To further understand the factors affecting PDX engraftment, growth kinetics, and genomic stability, we also analyzed PDXs by histopathological and molecular analysis. Histopathological results of 4 markers tested, TTF1, P40, Ki67, and PDL-1, showed that biopsy original biomarkers were preserved mainly in early PDX passages. In contrast, those biomarkers were only partially retained in later passages. TTF1 expression, a diagnostic marker for adenocarcinoma, was consistent primarily in early and intermediate PDX passages, while P40 positivity, a marker for squamous cell differentiation, varied throughout generations of PDXs. P40 results may be explained by the focal expression of P40 in the tumor tissue, as previously suggested by others [28]. Ki67 expression levels, a proliferation marker related to poor prognosis [29, 30], seemed to change throughout the passages. However, it is worth noting that recent work has questioned its predictive impact on response to treatment [30]. PDL-1 expression levels were consistent throughout the passages. Although PDL-1 expression levels were stable in our study, heterogeneous expression and lack of uniformity were reported depending on the scoring algorithms. These differences in reported PDL-1 expression levels should be noticed since they may hamper its utility and clinical significance [31].

Our analysis also found tumors that showed a high discrepancy between the original biopsy and early and late PDX passage samples. PDX37 is a prime example of a tumor that completely lost its histopathological characteristics in the advanced passage (P7) and transformed into a poorly differentiated malignant tumor with no specific differentiation features (Fig. 4).

Molecular profiling results using WES analysis followed the same pattern as the histopathological analysis and revealed discrepancies between the early and late PDX passages. Mutational changes were seen primarily in advanced PDX passages, although we also observed low tumor stability in early PDX passages, demonstrating unexpected changes in clinically relevant driver mutation (Fig. 5). The genomic stability of NSCLC PDX models remains debatable, and studies have shown that PDX models from NSCLC recapitulate the genomic landscape in early PDX passages [11, 32]. In contrast, others have demonstrated fundamental mutational changes between PDXs and original match biopsies [21, 26]. The cause for these molecular discrepancies was suggested to be linked to several factors, including clonal dynamics, proceeded by PDX serial passaging [26, 32–35], mixing of murine and human–stromal tissue [36, 37], and the tumor microenvironment (TME) [38, 39]. NSCLC PDX models have shown that the TME is drastically changed and replaced by murine stroma, leading to murine-specific clonal selection [22], and that murine stromal cells actively adopt a human-like phenotype in early generations [40]. Clonal evolution in advanced PDX passages becomes more deterministic rather than stochastic, probably due to specific TME factors that interfere with intratumoral heterogeneity (ITH) [27, 41].

Our results from several selected PDXs showed that changes in tumor molecular landscapes were observed mainly in PDXs collected from patients with aggressive tumor behavior and that most detected variants occurred in low frequencies in all PDX samples. However, variants that occurred both in the early and late passages of the same PDX were found to be of higher frequencies, suggesting these variants compose the cancer-associated mutations that gained dominance and led to clonal evolution. Comparison to clinically relevant mutations detected by NGS before tumor resection found both preserved and new gene alternation. In our cases, TP53 was retained in both early and late PDX passages. EGFR mutation, which is clinically highly important for identifying patients eligible for treatment with TKIs [42], was retained in the early PDX passages but revealed a different nucleotide variant in the late passage. The specific EGFR mutation significantly impacts not only treatment decisions but also the response to treatment and the mechanisms of resistance that are likely to develop (see [42, 43]).

Despite our best efforts, our study is subject to several limitations. These include our cohort sample size, the lack of complete OMICS analyses, high diversity of patients’ demographic and clinical background, such as disease stage, tumor histological type, and treatment history. Moreover, we were not able to investigate the effect of human-derived cells such as tumor infiltrating lymphocytes (TILs) and fibroblasts, on our PDX initial and sequential engraftment success.

Nevertheless, we show that multiple factors can affect PDX engraftment success and growth rate, and these should be considered when developing and using NSCLC PDX models. We demonstrated that variability between the PDX and the parental tumor was increased in later passages, a phenomenon likely attributed to ITH changes and clonal selection; however, this has yet to be proven. Our data indicate that PDX models can serve as valuable preclinical tools for NSCLC research, although it is essential to recognize the model's limitations of these models and to cautiously use them for predicting patient treatment response, identifying biomarkers, and early-stage clinical trials. We emphasize the need for additional research to definitively establish the clinical relevance of our NSCLC PDX model and its potential in advancing patient care and treatment.

### Supplementary Information

Below is the link to the electronic supplementary material.

Supplementary file1 (DOCX 27 KB)

## Data Availability

The datasets generated during and/or analyzed during the current study are available from the corresponding author upon reasonable request and upon ethical approval.
