# Supplementary material for: Personalizing non-small cell lung cancer treatment through patient-derived xenograft models: preclinical and clinical factors for consideration
Source: Clin Transl Oncol. 2024 Mar 29;26(9):2227–39. doi: 10.1007/s12094-024-03450-3 (PMC11333550; doi:10.1007/s12094-024-03450-3)
Supplement: Supplementary file 1 — Supplementary file1 (DOCX 27 KB) [file 12094_2024_3450_MOESM1_ESM.docx]

**Table S1. NSCLC patient, tumor, and xenograft characteristics.** A detailed description of histological, immunohistochemical, and molecular characteristics for each NSCLC patient whose tumor was engrafted in NSG-SGM3 mice to create a PDX model. PDX tumor take rate indicates the engraftment rate of the PDX within three engrafted NSG-SGM3 mice.

| **PDX** | **Patient age** | **Patient gender** | **Smoking history** | **Sample source (D/S/R)*** | **Histologic diagnosis** | **Stage** | **Genetic alterations** | **Treatment history** | **Patient survival  (months)** | **PDX engraftment date** | **PDX take rate** |
| --- | --- | --- | --- | --- | --- | --- | --- | --- | --- | --- | --- |
| PDX1 | 63 | M | no | lung (S) | adenocarcinoma | IV | FMI KRAS G12A STK11 E33fs*14 TP53 R248L | 12.2016 - cis/pem/bev x 5 with significant PR 3.2017 - alimta + keytruda with metabolic CR (stop alimta at 9.2017 and cont keytruda) | 47 | 1.8.19 | 3/3 |
| PDX2 | 57 | F | no | lung (S) | adenocarcinoma | IV | EGFR amplification, E746_L747>VP MDM2 amplification MYC amplification CDKN2A loss FRS2 amplification | 2.2018 - carb/alimta/ keytruda 3.2018 - tarceva with mixed response | 14 | 16.2.20 | 3/3 |
| PDX3 | 67 | M | yes | lung (S) | adenocarcinoma | IV | ERBB2, ex20 mutation; PDL1 25-49% ERBB2 A775_G776insYVMA | 8.2016 - cis/pem X 2  10.2016 - keytruda X 10 with SD PR best response 5.2017 - keytruda + ipilimumab (1 mg/kg Q4 wks) X 7 PR best response but liver single PD lesion for SBRT (cont keytruda) 10.2017 - SBRT to liver single lesion and cont keytruda 4.2018 - re-FDG uptake of liver mets (was radiated) cont keytruda till 7.2018 | 17 | 3.11.19 | 1/3 |
| PDX4 | 48 | F | yes | lung (D) | squamous cell carcinoma | IV | KRAS G12C/STK11 deletion exon 1/Myc amp/CDC73/CDKN2A/B/RAD21 amp/TP53 R196 | 03.19: Carbo/Tax/Keytru 05.19: keytru/carbo/Gemzar 07.19: Nivo/Ipi/Imiquimode 10.19: Anakinra/Opdivo, 1 2.19: Navelbine | 11 | 31.3.19 | 3/3 |
| PDX5 | 51 | F | yes | lung (S) | mesothelioma | IV | BAP1 Q684, CDKN2A/B, NOTCH3 R1893, MSS 4mut/Mb, ATM | 03.18: Carbo/Alimta/Avastin 04.19: keytru/Ipi 02.20: keytru 3.20: adding Lenvatinib 10mg (29c) with PR (7/4) on physical examination (30/04/20) 6.20: increased Lenvatinib to 14mg daily and adding Gemzar D1 Q21 due to mixed response | 44 | 3.4.19 | 3/3 |
| PDX6 | 57 | M | yes | lung (R) | neuroendocrine carcinoma | IV | PTEN, TP53, RB1 | 04.19: Cis/Alimata/Keytruda 01.20: keytruda | 20 | 27.8.19 | 0/3 |
| PDX7 | 74 | F | no | lung | adenocarcinoma | Ia | TTF1-neg, ER-neg, PDL1 1-49%, BRAC1 E23fs, KRAS G12V, CDKN2A deletion ex1 | 05.18: Carbo/Alimta | 57 | 3.9.19 | 0/3 |
| PDX8 | 59 | F | yes | lung | adenocarcinoma | Ia | EGFR/ALK/ROS wt, PDL1=0 | 03.19: Carbo/Alimta/Keytruda 02.20: Keytruda | 47 | 11.9.19 | 0/3 |
| PDX10 | 77 | F | no | lung (D) | adenocarcinoma | Ia | not tested | no treatment | 41 | 5.11.19 | 0/3 |
| PDX11 | 68 | M | yes | lung (D) | squamous cell carcinoma | Ia | not tested | 05.19: Carbo/Avraxane/Atezolizumab 01.20: Atezolizumab | 40 | 15.10.19 | 0/3 |
| PDX12 | 61 | M | yes | lung (D) | adenocarcinoma | IIb | TTF1 +, EGFR L858R (3.8%), NFE2L2 T80A, TP53 P278L, ALK ROS1 (-), PDL1<1% | 10.19: Afatinib | 5.5 | 27.11.19 | 0/3 |
| PDX13 | 60 | F | yes | lung (R) | adenocarcinoma | IV | BRAF V600E | 05.19: Cis/Navelbine 02.20: Carbo/Pemet/Keytruda | 44 | 27.11.19 | 0/3 |
| PDX14 | 80 | M | yes | lung (D) | squamous cell carcinoma | Ia | not tested | 02.20: CELCOX, ZALDIAR | 36 | 27.11.19 | 3/3 |
| PDX15 | 57 | M | yes | lung (D) | mucinous adenocarcinoma | Ib | not tested | no treatment | 36 | 3.12.19 | 0/3 |
| PDX16 | 53 | M | yes | brain (D) | adenocarcinoma | IV | not tested | no treatment | 21 | 8.12.19 | 2/3 |
| PDX17 | 57 | M | yes | lung (D) | adenocarcinoma | Ia | not tested | no treatment | 36 | 10.12.19 | 0/3 |
| PDX19 | 63 | M | no | brain (D) | adenocarcinoma | IV | not tested | 03.20: Alimta/Carbo/keytruda 04.20: Alimta/Carbo/keytruda/Zomera | 16 | 12.12.19 | 0/3 |
| PDX21 | 54 | M | yes | spine (S) | adenocarcinoma | IV | EGFR/ALK/ROS (WT) 4 mut/mb PDL1>50% 4.2017: CDKN2AB loss; MSI stable 9 Mut/Mb PDL1 (1-25%) | 2.2016 - cis/pem/bev x 4 with CR 1.2017 - atezolizumab x 5 4.2017 - cis/pem/keytruda x 3 with PR 6.2017 - definitive radiation (60GY)with cont cis/alimtax2 7.2017 - cont keytruda with metabolic CR 3.2018 - keytruda + adding carbo/alimta x 4 --> keytruda/alimta | 48 | 17.12.19 | 0/3 |
| PDX22 | 77 | M | yes | lung (D) | squamous cell carcinoma | Ia | not tested | no treatment | 10.5 | 18.12.19 | 0/3 |
| PDX23 | 58 | M | yes | lung (D) | adenocarcinoma | IV | PDL1<1%, EGFR WT, ALK neg, ROS1 neg | 20.1.20 - carbo alimta keytruda | 2 | 18.12.19 | 3/3 |
| PDX24 | 63 | M | no | lymph node (S) | adenocarcinoma | IV | KRAS G12A, STK11 E33fs, TP53, NF1 P427fs , NF1 MSS 3.3 mut/mb | 12.16 - cis/pem/bev x5 with PR 3.2017 - Alimta + keytruda with metabolic CR 5.2018 - keytruda rechallenge with PD 7.2018 - keytruda + carbo + alimta X4 with sig response 5.2019 - nivolumab/ ipilimumab x 4 with PD 1.2020 - keytruda + lenvatinib | 47 | 23.12.19 | 0/3 |
| PDX25 | 54 | F | yes | lung (R) | adenocarcinoma | IIb | ROS1+ | 08.19: Crizotinib, 11.19: Brigatinib, Bran 01.20 Lorlatinib | 25 | 5.1.20 | 0/3 |
| PDX26 | 72 | F | yes | lung (R) | adenocarcinoma | IV | KRAS G12C, TP53, BRAF G596C, KEAP1 G429C, 7.4 Mut/Mb MSS PDL1- 30% | 07.15: Carbo/Alimta/Avastin,  10.15: Nivo 03.20: Taxotere | 58.5 | 15.1.20 | 0/3 |
| PDX27 | 54 | F | yes | lung (S) | adenocarcinoma | IV | EGFR ex19 +, ALK/ROS wt | 02.19: Tagrisso 02.20: Carbo/Alimta/keytru | 16.5 | 23.1.20 | 0/3 |
| PDX28 | 76 | F | no | lung (S) | adenocarcinoma | IV | not tested | 6.2019 - chemo |  | 26.1.20 | 1/3 |
| PDX29 | 61 | F | yes | brain (D) | adenocarcinoma | IV | EGFR/BRAF/ALK/ROS -wt, PDL1>50% | 03.20: Carbo/Alimta/Keytru | 32.5 | 24.3.20 | 0/3 |
| PDX30 | 51 | M | yes | brain (D) | squamous cell carcinoma | IV | PDL1>50% | no treatment | 6.5 | 2.4.20 | 0/3 |
| PDX31 | 68 | M | yes | subcutan (S) | adenocarcinoma | IV | FMI 21 mut/mb; KRAS amp; RB1; STK11 (+); MDM2 amp | 1/2019 - keytruda + carbo/alimta X 5 1/2020 - continue keytruda alimta X 3 | 24 | 27.4.20 | 3/3 |
| PDX32 | 62 | M | yes | lymph node (S) | adenocarcinoma | IIIb | not tested | 4.20 - carbo/alimta X 2 with SD best response | 10 | 18.5.20 | 0/3 |
| PDX33 | 63 | M | no | lung | adenocarcinoma | IIb | 7.2019 tempus: egfr ex19 (MAF 33%), TP53, EGFR amp , 3.3. mut/mb MSS 7.2019 cfDNA: EGFR ex19 0.4%, NTRK I638V 0.1%, ATM L1794R 0.1%, CCNE 1.2020 cfDNA: EGFR ex19 0.08%, ATM L1794R 0.2%, TP53, total tDNA 0.3% | 1.2017 - multiple SRS to brain (10/14 lesions) 1.2017 - tagrisso 12.2017 - brain PD --> tagrisso 160 mg with PR best response 5.2019 - tagrisso 80 mg 7.2019 - systemic PD, brain SD  7.2019 - Cis/alimta x 3 + alternate tagrisso 80 mg with significant PR --> Alimta x 4 + (tagrisso alternate) | 70 | 27.5.20 | 3/3 |
| PDX34 | 42 | M | yes | lung | adenocarcinoma | Ia | PDL1>50%, EGFR/ALK/ROS1/RAF/KRAS wt; TP53 FMI: KRAS G12C; TP53 | 8.2019 - SRS left cerebellar X 1 9.2019: cis/alimta/keytruda X 4 --> alimta/keytruda X 2 with significant response  1.2020: keytruda (single agent) X 4 | 40 | 1.6.20 | 1/3 |
| PDX35 | 63 | M | no | lymph node (S) | adenocarcinoma | IV | 02.20: ADC, No mutation 2.2020: foundation1 - CCND3 amp, DAXX C629fs*16, NKX2-1 amplification - equivocal, TP53 F212fs*34, VEGFA amplification. Microsatelite status - MS stable, tumor mutational burden - 9 muts/mb | 03.20: Alimta/Carbo/keytruda 04.20: Alimta/Carbo/keytruda/Zomera 6.20: disease progression | 18 | 15.6.20 | 2/3 |
| PDX36 | 59 | F | no | lung | adenocarcinoma | IIb | ALK+ | unknown | 18 | 22.6.20 | 3/3 |
| PDX37 | 69 | M | yes | lymph node (S) | adenocarcinoma | IV | 6.2020 - EGFR L858R (40.1%), EGFR amp (++), TP53 S183 (5.1%), MYC amp (++) | 7.2019 - Tagrisso + palliative RT to rib 6 with PR (brain + systemic) 2.2020 - For left iliac crest palliative radiation 6.2020 - RUL: Left Pelvic PD; right adrenal PD 6.2020 - left iliac radiation 6.2020 - for carbo/alimta and cont tagrisso | 2 | 22.6.20 | 3/3 |
| PDX38 | 77 | F | no | lung (D) | adenocarcinoma | II | not tested | no treatment | 29 | 23.6.20 | 3/3 |
| PDX39 | 66 | M | yes | subcutan (S) | adenocarcinoma | Ia | FMI: RET (+) rearrangement intron 10; high TMB 75 mut/mb; MSS; PDL1<1% 4.2020 - NGS (ichilov): BRAF V600E' no RET was detected although RNA seq | 12.3.2020 - systemic PD 4.2020 - for C4-C6 radiation (assuta) 4.2020 - carbo/alimta/ keytruda X 2 with mostly SD, D3/D5 FDG lesions | 56 | 9.7.20 | 3/3 |
| PDX40 | 75 | M | yes | lymph node (S) | neuroendocrine carcinoma | IV | 3.2020 - cfDNA guardant: BRCA2 K216fs, MET amplification 7.2020 - RB1 p.1550* Stop gain-LOF, TP53 c.920-2A>G Splice region variant LOF, RASA1 p.R679* stop gain-LOF, FH p.G9fs Frameshift LOF, KEAP1 Copy number loss | 3.2020 - VP16 80% + carbo AUC 4 + durvalumab (mild PR only metabolic) | 14 | 13.7.20 | 3/3 |
| PDX41 | 73 | F | no | lung | adenocarcinoma | Ia | EGFR L858R 44% TP53 Glu271_Arg272del 31% | NeoAdjuvant study implantation under Tagrisso. | 32 | 12.8.20 | 0/3 |
| PDX42 | 73 | F | no | liver (S) | adenocarcinoma | IV | 3.2016 - EGFR ex21 stage IIIA NSCLC 5.2018 - EGFR L858R + L833V (T790m-) 10.2020 - MET amp 1.15 EGFR L858R MAF 61% and L833V MAF 61% | 4.2016 -carbo/alimta & 60 GY 5.2018 - tarceva with PR 1.2019 - carbo/alimta +keytruda till 11.2019 2.2020 - oral navelbine 1.2021 - recommended for tagrisso | 25 | 4.11.20 | 3/3 |
| PDX43 | 43 | F | yes | lung (D) | adenocarcinoma | IV | not tested | no treatment | 25 | 4.11.20 | 3/3 |
| ****D=diagnostic biopsy, S=surgery, R=recurrence biopsy*** | | | | | | | | | | | |
